# The microbiota-gut-brain axis and central nervous system diseases: from mechanisms of pathogenesis to therapeutic strategies

**DOI:** 10.3389/fmicb.2025.1583562

**Published:** 2025-06-13

**Authors:** Jie Xu, Yi Lu

**Affiliations:** Key Laboratory of Modern Chinese Medicine Preparation of Ministry of Education, Jiangxi University of Traditional Chinese Medicine, Nanchang, China

**Keywords:** microbiota-gut-brain axis, central nervous system diseases, gut microbiota, pathogenesis, treatment

## Abstract

The gut microbiota plays a crucial role in metabolic processes associated with host brain function. Emerging research is progressively uncovering the intricate and multifaceted relationship between the gut and the brain. The gut microbiota significantly influences immune responses, secondary metabolism, and symbiosis with the host, thereby facilitating the production of essential metabolites, neurotransmitters, and other neuroactive compounds that impact the development and treatment of central nervous system disorders. This article delineates the communication pathways and mechanisms linking the microbiota, gut, and brain, providing a comprehensive overview of current research on how the gut microbiota affects nervous system function. Furthermore, it examines factors that can alter the gut microbiota and influence metabolite profiles, as well as current intervention strategies aimed at enhancing gut-brain communication, mitigating adverse triggers that disrupt the gut microbiota, and minimizing neuro-pathological changes.

## 1 Introduction

Central nervous system diseases (CNSD) pose a significant threat to human health and exhibit an alarmingly high incidence rate globally. This category of diseases includes conditions such as stroke, neurodegenerative disorders (e.g., Alzheimer’s disease and Parkinson’s disease), spinal cord injury, mood disorders (e.g., anxiety and depression), and cerebral infarction (Lei et al., 2024). The complexity of CNSDs is not only reflected in their diverse types but also in their multifaceted etiology, involving genetic factors, environmental influences, and lifestyle choices (Linnerbauer and Rothhammer, 2020). For instance, the onset of Alzheimer’s disease is associated with specific gene mutations, chronic inflammation, and long-term unhealthy habits such as physical inactivity and a high-sugar diet (Lardelli et al., 2024); while depression is closely linked to psychological stress, neurotransmitter imbalances, and alterations in brain neural plasticity (Luscher et al., 2023). The investigation into organ axes is reshaping the contemporary medical framework for comprehending diseases. Through their multi-dimensional regulatory mechanisms, these axes provide novel targets for the precise treatment of complex diseases, including the central nervous system-spleen axis (Khan et al., 2021), liver-gut axis (Wang et al., 2024b), liver-spleen axis (Tarantino et al., 2021), lung-gut axis (Li J. et al., 2024), kidney-gut axis (Yu D. et al., 2024), and brain-gut axis (Caldarelli et al., 2024). The transition from “single-organ treatment” to “axis-based network regulation” represents a paradigm shift that has unveiled innovative strategies for the prevention and management of major diseases, such as burns, non-alcoholic fatty liver disease, and neurodegenerative disorders. In recent years, the microbiota-gut-brain axis (MGBA) has emerged as a burgeoning research field and has become a focal point in neuroscience, offering novel insights into the study and treatment of CNSDs. The MGBA constitutes a complex bidirectional communication network that involves interactions between gut microbiota, the intestinal barrier, the immune system, the vagus nerve, the enteric nervous system, and the central nervous system (Loh et al., 2024). Gut microbiota plays a critical role not only in host digestion and nutrient absorption but also in immune regulation and the production of metabolic products. Accumulating evidence suggests that dysbiosis of gut microbiota is closely associated with the pathogenesis and progression of various CNSDs. For example, changes in the composition and function of gut microbiota have been observed in conditions such as Alzheimer’s disease, Parkinson’s disease, and depression (Liu et al., 2023).

The microbiota, comprising bacteria, viruses, fungi, and other microorganisms that coexist with the host, is recognized as an integral component of human physiology and plays a critical role in maintaining health and influencing disease progression (Arifuzzaman et al., 2024). The gut microbiota, the largest microbial reservoir in the human body, begins to colonize during infancy and evolves alongside the host throughout life (Lu et al., 2024). Recent studies indicate that the gut microbiota and its metabolites are crucial for neurodevelopment, neurodegenerative diseases, and other neurological disorders (McCallum and Tropini, 2024). Research has confirmed that during sepsis, the gut microbiota undergoes pathological alterations, potentially leading to the translocation of gut bacteria to the brain, which may contribute to the pathogenesis of sepsis-associated encephalopathy (SAE) (Fang et al., 2022). Among the microbiota, certain gerena such as *Lactobacillus* and *Bifidobacterium* are considered beneficial and are associated with enhanced intestinal barrier function. Multiple studies have demonstrated that in SAE mouse models, the richness and diversity of the gut microbiota are significantly reduced, and the populations of beneficial bacteria like *Lactobacillus* and *Bifidobacterium* are markedly diminished, suggesting that the gut microbiota plays a significant role in the development of SAE (Li et al., 2018; Xi et al., 2022). Further research has revealed that pre-sepsis alterations in the gut microbiota can lead to the expansion of pathogenic microorganisms, trigger robust inflammatory responses from the immune system, and reduce the production of beneficial microbial metabolites, thereby increasing susceptibility to sepsis (Zhang M. L. et al., 2022). These changes may impact the function of terminal organs, including the brain. In-depth exploration of the connection between the MGBA and central nervous system diseases, from pathogenesis to therapeutic strategies, holds promise for opening new avenues in CNSD treatment, developing more effective interventions, and improving patient outcomes.

## 2 Microbiota-gut-brain axis

In the 1840s, William demonstrated through experiments that emotional states could influence digestion speed, suggesting that the brain affects intestinal function and indicating an axis of communication between the brain and the gut (Browning and Travagli, 2014). A substantial body of research has demonstrated that the central nervous system (CNS) directly modulates intestinal function via the autonomic nervous system (ANS) and collaborates with the enteric nervous system (ENS) to maintain intestinal homeostasis (Dicks, 2023; Griffiths et al., 2024; He et al., 2024). The CNS can also activate the hypothalamic–pituitary–adrenal axis (HPAA), promoting the release of norepinephrine and adrenocorticotropic hormone, which can lead to alterations in the intestinal microbiota and gut function (Kasarello et al., 2023; Tiwari and Paramanik, 2025). Additionally, it can directly stimulate the immune system to release inflammatory cytokines, causing intestinal inflammation, disrupting intestinal barrier integrity, and leading to dysbiosis of the gut microbiota (Levard et al., 2024). Subsequent studies have further revealed that changes in the gastrointestinal tract, including altered gut function and compromised intestinal barrier, can contribute to complex CNS disorders (El-Hakim et al., 2022; Pellegrini et al., 2023). Intestinal signals can be transmitted to the brain via the ENS and vagus nerve, influencing brain function and participating in disease regulation. The HPAA can also be activated by inflammatory factors produced by gastrointestinal lesions, leading to increased cortisol secretion and subsequent modulation of brain function (Pedraz-Petrozzi et al., 2023). Therefore, it is evident that the CNS regulates intestinal function and maintains gastrointestinal homeostasis through neural and endocrine pathways. Conversely, intestinal information can reach the CNS via multiple pathways to modulate brain function. This bidirectional and complex signaling pathway is referred to as the brain-gut axis.

The MGBA, an extension of the gut-brain axis, further emphasizes the critical role of gut microbiota in the bidirectional communication between the gut and the brain, forming a more intricate network (Wang Y. et al., 2023). The gut microbiota is highly diverse and abundant, participating in numerous physiological processes. On the one hand, gut microbiota influences brain function through their metabolic products. They produce short-chain fatty acids (SCFAs) and neurotransmitters such as serotonin, dopamine, and γ-Aminobutyric acid (GABA) (Mi et al., 2023). SCFAs can modulate blood–brain barrier permeability, affect brain immune responses, and regulate gene expression in neurons. Approximately 90% of serotonin is synthesized in the gut, and changes in its levels can impact mood and cognition. When gut microbiota dysbiosis leads to reduced serotonin synthesis, it may contribute to mood disorders such as depression and anxiety (Xiong et al., 2024). On the other hand, gut microbiota can influence brain function via the immune system and the vagus nerve. Dysbiosis of gut microbiota can activate the immune system, leading to the production of inflammatory cytokines. These cytokines can affect brain neural function through the bloodstream or by directly acting on the vagus nerve, and are associated with the pathogenesis and progression of neurodegenerative diseases such as Alzheimer’s disease and Parkinson’s disease (Cui et al., 2024).

## 3 Gut microbiota promotes brain health

### 3.1 The interaction between gut microbiota and gastrointestinal motility disorders

Disorders of the gut-brain axis, also referred to as functional gastrointestinal disorders, represent a group of conditions characterized by chronic gastrointestinal symptoms such as abdominal pain, nausea, bloating, constipation, and diarrhea, in the absence of identifiable structural or inflammatory pathology (Kraimi et al., 2024). Studies have demonstrated that gut microbiota and gastrointestinal motility are interrelated and mutually influence each other both physiologically and pathologically. This interaction occurs through multiple pathways, including the enteric nervous system (enteric neurons and glial cells), the intestinal barrier (enterochromaffin cells), and the intestinal immune system (macrophages in the intestinal muscular layer) (Shah et al., 2024; Marques de Souza et al., 2025).

#### 3.1.1 Gut microbiota modulates gastrointestinal motility via the enteric nervous system

Studies utilizing germ-free (GF) neonatal mice as experimental models have demonstrated that gut microbiota may directly influence the postnatal development of the ENS (Ganz and Ratcliffe, 2023). Specifically, gut microbiota may regulate gastrointestinal motility by altering the number of neurons and the proportion of different neuron subtypes within the ENS (Griffiths et al., 2024). Recent single-cell sequencing analyses indicate that gut microbiota dysbiosis in patients with congenital megacolon is closely associated with abnormal expression of key ENS developmental genes, such as RET, suggesting that microbiota may regulate intestinal ganglion formation by modulating neural crest cell migration (Zhou et al., 2024). In achalasia, reduced abundance of *Rhodobacter* leads to excessive LPS secretion, which activates the TLR4/MYD88/NF-κB signaling pathway, resulting in degeneration of inhibitory neurons (Geng et al., 2023). Caetano et al. (2023) confirmed that GPR41 is expressed in interstitial neurons of the intestine, and butyrate can bind to GPR41 to mitigate neuronal loss and glial hyperplasia induced by experimental ulcerative colitis, while also improving morphological damage to intestinal tissue.

Early studies posited that enteric glial cells (EGCs) primarily provided mechanical support to enteric neurons; however, accumulating evidence reveals that as the most abundant cell type in the ENS, EGCs play critical roles in maintaining intestinal immune homeostasis, preserving intestinal barrier integrity, and regulating gut microbiota composition (Li et al., 2024b; Zhang C. et al., 2024; Brown et al., 2025). Montalbán-Rodríguez et al. (2024) further demonstrated that in Parkinson’s disease (PD) patients, EGCs exhibit activation and reactive gliosis, potentially triggering immune/inflammatory responses via Toll-like receptors (TLRs), promoting α-synuclein aggregation and neurodegeneration. Abnormal activation of EGCs may be reversed, and neuroinflammation alleviated, through interventions such as tryptophan-2,3-dioxygenase inhibitors, nutritional supplementation, or physical exercise.

#### 3.1.2 The gut microbiota modulates gastrointestinal motility by influencing the intestinal barrier

Impaired intestinal barrier function represents a critical pathological basis for motility disorders (Grover et al., 2025). Probiotics, such as *Lacticaseibacillus paracasei* (basonym *Lactobacillus paracasei*), enhance the mechanical integrity of the intestinal mucosa by upregulating the expression of tight junction proteins (claudin-1, ZO-1, Occludin), thereby reducing LPS translocation across the epithelium (Su et al., 2024). Conversely, exposure to antibiotics or a high-sugar diet can disrupt the mucus layer, promoting the overgrowth of opportunistic pathogens. For instance, in patients with diabetic gastroparesis, long-term hyperglycemia and other factors often lead to a significant reduction in the expression of the SCF/c-kit signaling pathway. This results in a decreased number of interstitial cells of Cajal (ICCs) and damage to their ultrastructure, facilitating the translocation of harmful substances such as bacteria and endotoxins into the systemic circulation, triggering enteric neuroinflammation and inhibiting motilin secretion (Zheng X. et al., 2024). Multiple studies have demonstrated that modulating the gut microbiota can restore intestinal barrier function, thereby alleviating gastrointestinal-related symptoms (Chen et al., 2023; Wang D. et al., 2023; Jia X. et al., 2024; Zeng H. et al., 2024; Zhang et al., 2024a).

#### 3.1.3 The gut microbiota modulates gastrointestinal motility by influencing intestinal immune function

The gut microbiota can activate mucosal immune cells, including macrophages, which in turn influence the composition and function of the gut microbiota (Wang et al., 2024a). Muscularis macrophages (MMs), located in the intestinal muscular layer, are closely associated with enteric neurons (EN), interstitial cells of Cajal (ICCs), and smooth muscle cells (SMC). Consequently, the anti-inflammatory properties of MMs play a critical role in the development and maintenance of the gastrointestinal neural network (Zhou et al., 2023a; Chikkamenahalli et al., 2024). The transition of MMs from an anti-inflammatory to a pro-inflammatory state may contribute to the inflammatory mechanisms underlying various gastrointestinal disorders, including functional conditions such as diabetic gastroparesis, postoperative intestinal obstruction, and irritable bowel syndrome, as well as organic diseases like inflammatory bowel disease (Choi et al., 2024). Research indicates that muscularis macrophages (MMs) primarily modulate intestinal motility by secreting bone morphogenetic protein 2 (BMP2), which activates BMP2-related receptors on enteric neurons and influences neuronal activity. Moreover, the gut microbiota can interact with MMs, thereby regulating gastrointestinal motility, with BMP2 likely playing a critical role in this interaction (Li et al., 2024a). Furthermore, studies have shown that optimizing the structure of the gut microbiota, enhancing the abundance of beneficial bacteria such as *Muribaculaceae*, reducing the number of MMs in colonic tissue, downregulating pro-inflammatory factor expression, and upregulating BMP2 secretion by MMs can enhance gastrointestinal motility, inhibit neuronal loss, and improve slow transit constipation in mice (Ren et al., 2022).

### 3.2 Interaction between microbiota and neurotransmitters

#### 3.2.1 Synthesis and regulation of serotonin

Serotonin (5-hydroxytryptamine, 5-HT) is an essential neurotransmitter that plays a critical role in regulating emotions, cognition, sleep, and other physiological processes (Bremshey et al., 2024). Approximately 90% of serotonin is synthesized in the gut, where the gut microbiota plays an indispensable role in this process (Sanidad et al., 2024). Certain bacteria in the gut, such as *Escherichia coli*, *Lactobacillus*, and *Bifidobacterium*, can participate in the metabolism of tryptophan, the precursor to serotonin synthesis (Ala, 2022). For example, *Limosilactobacillus reuteri* (basonym *Lactobacillus reuteri*) is capable of converting tryptophan into 5-hydroxytryptophan via specific metabolic pathways, thereby facilitating serotonin synthesis (Xie et al., 2020); *Lactiplantibacillus plantarum* (basonym *Lactobacillus plantarum*) can stimulate serotonin secretion by host enterochromaffin cells (Lu et al., 2021); *Bifidobacterium breve*, *Bifidobacterium longum*, and *Pediococcus acidilactici* are able to enhance the production of 5-hydroxytryptophan and 5-HT in the intestine, thus promoting systemic 5-HT circulation (Tian et al., 2023); *Escherichia coli*, *Akkermansia muciniphila*, and *Faecalibacterium prausnitzii* contribute to improving intestinal serotonin balance through the regulation of serotonergic genes (Yaghoubfar et al., 2020, 2021; Olivo-Martínez et al., 2024). Studies have shown that in germ-free mouse models, the absence of gut microbiota leads to significantly lower serotonin levels compared to normal mice. However, when these germ-free mice are transplanted with gut microbiota, their serotonin levels increase significantly (Pan et al., 2025). This experiment provides strong evidence of the significant impact of gut microbiota on serotonin synthesis. It is important to highlight that the gut microbiota not only modulates serotonin synthesis but also plays a critical role in regulating its metabolic balance through the modulation of degrading enzymes. For example, *Escherichia coli* secretes β-glucuronidase, which facilitates the conjugation of serotonin with glucuronic acid to form the water-soluble metabolite 5-hydroxytryptophan glucuronide (5-HTG), thereby enhancing its excretion (Ala, 2022). Furthermore, *Clostridium scindens* upregulates monoamine oxidase A (MAO-A) activity, promoting the oxidative deamination of serotonin into the inactive metabolite 5-hydroxyindoleacetic acid (5-HIAA) (Li et al., 2025). In individuals with anxiety disorders, dysbiosis of the gut microbiota is frequently associated with elevated levels of β-glucuronidase and MAO-A activity, leading to accelerated serotonin degradation (Simpson et al., 2024). Supplementation with specific probiotics, such as *Lacticaseibacillus rhamnosus* (basonym *Lactobacillus rhamnosus*), *Lactobacillus acidophilus*, and *Lactiplantibacillus plantarum* (basonym *Lactobacillus plantarum*), not only facilitates the metabolic conversion of tryptophan to serotonin but also suppresses MAO-A activity via the secretion of short-chain fatty acids (SCFAs), thereby enhancing serotonin bioavailability through dual mechanisms (Tsai et al., 2023). These findings underscore the pivotal role of the gut microbiota in the pathophysiology of mental health disorders, including depression, through the bidirectional regulation of synthetic and degrading enzymes.

#### 3.2.2 Regulation of γ-aminobutyric acid

γ-aminobutyric acid (GABA) is the primary inhibitory neurotransmitter in the brain and plays a critical role in maintaining neural excitability balance. The gut microbiota significantly influences GABA synthesis and metabolism (Braga et al., 2024). Certain gut bacteria, such as *Clostridium butyricum*, can modulate the expression of GABA-related genes in intestinal epithelial cells and immune cells through SCFAs, including butyric acid, thereby affecting GABA synthesis and release (Liu Z. et al., 2024). In animal studies, mice administered probiotic preparations rich in *Clostridium butyricum* exhibited significantly increased brain GABA levels and reduced anxiety-like behaviors (Zheng M. et al., 2024). These findings suggest that the gut microbiota can influence central nervous system disorders, such as anxiety, by regulating GABA levels. Additionally, an imbalance in the gut microbiota has been closely linked to abnormalities in the GABA system in epilepsy patients. Research indicates that a reduction in certain beneficial bacteria in the intestines of epilepsy patients leads to insufficient GABA synthesis and heightened neural excitability, exacerbating the frequency and severity of epileptic seizures (Dahlin et al., 2024). By modulating the gut microbiota, it may be possible to improve GABA system function and alleviate symptoms in epilepsy patients.

### 3.3 Interaction between the microbiota and neurotransmitter receptors

#### 3.3.1 The impact of the microbiota on 5-HT receptors

5-HTRs are critical mediators of serotonin signaling in both the CNS and gastrointestinal tract. Emerging evidence indicates that gut microbiota and their metabolites directly modulate 5-HTR expression and function through multiple pathways (Dutta Gupta et al., 2023). For instance, microbial metabolites such as SCFAs and tryptophan derivatives can act as ligands or signaling molecules that bind to or influence 5-HTRs (Buey et al., 2023). Butyrate, a SCFA produced by commensal bacteria like *Faecalibacterium prausnitzii*, has been shown to enhance the expression of 5-HT1A receptors in intestinal epithelial cells via histone deacetylase (HDAC) inhibition, thereby promoting anti-inflammatory and anxiolytic effects (Zhai et al., 2023). Research has demonstrated that the metabolite indole, which is associated with tryptophan metabolism, alleviates inflammatory responses by reducing the production of lipopolysaccharide-binding protein (LBP) and inhibiting the polarization of M1-type macrophages through activation of the intestinal 5-HT receptor HTR2B (Jiang L. et al., 2024). This elucidates the mechanism by which tryptophan modulates the 5-HT signaling pathway via the gut microbiota and its metabolites, thereby regulating intestinal immunity. Furthermore, enhancing the richness, diversity, and homogeneity of the gut microbiota, adjusting microbial composition, and promoting the production of SCFAs, particularly butyrate, can increase the expression of tryptophan hydroxylase (TPH) and bind to the 5-HT4 receptor (5-HT4R), thus improving intestinal motility and function (Liu Q. et al., 2024). A separate study revealed that the 5-HT4R antagonist GR 125487 may exacerbate dopaminergic neuron loss by inhibiting the JAK2/PKA/CREB signaling pathway, altering gut microbiota composition, and increasing reactive glial cells and neuroinflammation in the striatum (Cui et al., 2023). In germ-free mice, the absence of microbial signals results in reduced 5-HTR density and impaired serotonin signaling, effects that can be restored through probiotic supplementation (Pan et al., 2025). Clinically, patients with irritable bowel syndrome (IBS) exhibit altered gut microbiota composition, such as increased Pseudomonadota (basonym Proteobacteria) and decreased bifidobacteria, which correlates with abnormal overexpression of 5-HT3 receptors in colonic tissue. This overexpression enhances neuronal sensitivity to serotonin, contributing to symptoms such as pain and diarrhea (Merecz et al., 2023). These findings underscore the critical role of the gut microbiota in regulating 5-HTR expression and function through metabolic, immune, and epigenetic mechanisms, offering potential therapeutic strategies for related diseases.

#### 3.3.2 The impact of the microbiota on GABA receptors

The gamma-aminobutyric acid receptor (GABAR) is a major inhibitory neurotransmitter receptor in the brain, crucial for regulating neural excitability and maintaining neural functional balance (Shi W. et al., 2024). The gut microbiota can influence the expression and function of GABAR through multiple pathways. On the one hand, SCFAs, metabolic products of the gut microbiota, can regulate the expression of GABAR-related genes in the brain, thereby altering the quantity and function of GABAR (Shen et al., 2024). On the other hand, the gut microbiota can modulate the immune system to mitigate neuroinflammation-induced damage to GABARs (Mou et al., 2022). In epilepsy patients, abnormal GABAR function is a key factor contributing to epileptic seizures. Studies have shown that dysbiosis in the gut microbiota of epilepsy patients leads to reduced SCFA production, downregulation of GABAR-related gene expression, impaired GABAR function, and increased neural excitability, all of which contribute to seizure onset (Dahlin et al., 2024). By regulating the gut microbiota and enhancing SCFA production, it is possible to improve GABAR function, reduce neural excitability, and decrease the frequency and severity of epileptic seizures. Additionally, in patients with anxiety disorders, the impact of the gut microbiota on GABAR is closely linked to the occurrence of anxiety symptoms. Research indicates that alterations in the gut microbiota of anxiety disorder patients may lead to abnormal GABAR function, weakening the brain’s inhibitory effect of GABA, and thereby triggering anxiety (Ritz et al., 2024).

### 3.4 The impact of the microbiota on immune regulation

#### 3.4.1 Microbial metabolites regulate immune cell differentiation and function

Gut microbiota and their metabolites play a pivotal role in shaping the plasticity of immune cells via multiple mechanisms, including direct metabolic effects, modulation of immune signaling pathways, and epigenetic modifications. SCFAs, such as butyrate and propionate, which are generated through the fermentation of dietary fiber by gut microbiota, regulate immune cell differentiation via epigenetic mechanisms, such as histone deacetylase inhibition (Andrusaite et al., 2024). For example, butyrate enhances the generation of regulatory T cells (Tregs) while suppressing the activation of pro-inflammatory T help cell 17 (Th17) and Th1 cells (Liu J. et al., 2024). In patients with multiple sclerosis, reduced levels of Tregs are positively associated with decreased SCFA concentrations (Duscha et al., 2022). Furthermore, gut microbiota metabolize tryptophan into indole derivatives, such as indole-3-propionic acid, which modulate the balance between Tregs and Th17 cells by activating the aryl hydrocarbon receptor (AhR), thereby influencing immune tolerance and inflammatory responses (Huang et al., 2022; Jia D. et al., 2024). Indole-3-lactic acid, produced by *Lactiplantibacillus plantarum* (basonym *Lactobacillus plantarum*) during tryptophan metabolism, promotes the infiltration and activation of CD8^+^ T cells, contributing to the remodeling of the immune microenvironment (Zhang Q. et al., 2023). Beyond their fundamental physiological roles, bile acid metabolites derived from gut microbiota, such as deoxycholic acid and lithocholic acid and their derivatives, are also implicated in the differentiation and function of both innate and adaptive immune cells, including macrophages (Mac), dendritic cells (DC), myeloid-derived suppressor cells (MDSC), regulatory T cells (Treg), Breg cells, helper T cells (Th17, Th1, and Th2), CD8^+^ T cells, B cells, and NKT cells, thus maintaining local and systemic immune homeostasis (Su et al., 2023). Studies have shown that the composition of the gut microbiota in MS patients undergoes significant changes, characterized by a reduction in beneficial bacteria and an increase in harmful bacteria (Thirion et al., 2023). This microbial imbalance results in abnormal immune cell differentiation, excessive activation of Th1 and Th17 cells, and increased production of inflammatory factors, exacerbating neuroinflammation and tissue damage. By supplementing probiotics or performing fecal microbiota transplantation to regulate the gut microbiota, it is possible to restore normal immune cell differentiation, inhibit the activation of Th1 and Th17 cells, and alleviate inflammatory responses, thereby positively impacting the treatment of MS (Hasaniani et al., 2024).

#### 3.4.2 Microbial modulation of immune cell phenotypes

Gut-residing beneficial bacteria, such as *Bifidobacterium* and *Lactobacillus*, can activate dendritic cells via pattern recognition receptors (e.g., TLRs), thereby promoting their maturation and inducing the differentiation of anti-inflammatory regulatory Treg cells (Kedmi and Littman, 2024). Microbial metabolites play a critical role in regulating the polarization of macrophages from the pro-inflammatory M1 phenotype to the anti-inflammatory M2 phenotype (Solanki et al., 2023). For instance, butyrate supplementation has been shown to facilitate the transformation of macrophages from a pro-inflammatory state to an anti-inflammatory state in Parkinson’s disease (Karunaratne et al., 2020). Fecal microbiota transplantation (FMT) can restore gut microbial balance by reversing dysbiosis, inhibiting the upregulation of ERK and NF-κB signaling pathways, and suppressing microglial M1 polarization, thereby alleviating neural damage (Li et al., 2023). Furthermore, Gu et al. (2024) demonstrated that the intestinal mucosa exhibits a highly heterogeneous microenvironment, including distinct regions such as crypts, villi, and Peyer’s patches, which harbor significant differences in immune cell composition and function. Specifically, the crypt base is characterized by high expression of the Wnt signaling molecule R-spondin, which promotes Treg proliferation; the villus tip is enriched with prostaglandin E2 (PGE2) and retinoic acid (RA), driving the differentiation of Tregs into effector phenotypes; and dendritic cells (DCs) within the Peyer’s patch region maintain Treg suppressive function through the secretion of IL-10 and TGF-β.

#### 3.4.3 Dysbiosis of the intestinal microbiota and immune imbalance

The imbalance of the gut microbiota can lead to abnormal production of inflammatory factors, which can enter the CNS via the bloodstream or neural pathways, triggering neuroinflammation and thereby affecting CNS function (Guo et al., 2024). For instance, when harmful bacteria such as *Escherichia coli* overgrow in the gut, they produce large amounts of lipopolysaccharide (LPS), a potent inflammatory stimulant that activates the immune system and increases the release of inflammatory factors like tumor necrosis factor-α (TNF-α) and interleukin-6 (IL-6) (Di Vincenzo et al., 2024). In patients with Alzheimer’s disease (AD), neuroinflammation is a key pathological feature. Studies have shown that dysbiosis in the gut microbiota of AD patients leads to increased production of inflammatory stimulants such as LPS, which enter the brain through the bloodstream, activate microglia, and cause them to release large amounts of inflammatory factors, leading to neuronal damage and β-amyloid protein deposition, thereby accelerating AD progression (Marizzoni et al., 2020). By regulating the gut microbiota, reducing the number of harmful bacteria, and lowering the production of inflammatory factors, it is possible to alleviate neuroinflammation and delay the progression of AD (Varesi et al., 2022). Studies have demonstrated that supplementation with *Saccharomyces boulardii*, a probiotic yeast, effectively mitigates oxidative stress, inflammatory cytokines, and chemokines. This protective effect safeguards hippocampal neurons and ultimately reverses cognitive decline linked to gut microbiota dysbiosis (Roy Sarkar et al., 2021).

### 3.5 The impact of the microbiota on the blood–brain barrier

#### 3.5.1 Maintenance of the structure and function of the blood–brain barrier

The blood–brain barrier (BBB) is a critical protective structure for the CNS, restricting the entry of harmful substances and pathogens from the bloodstream into the brain and maintaining the stability of the brain’s internal environment (Shimizu and Nakamori, 2024). The gut microbiota influences the structure and function of the BBB through multiple pathways. On the one hand, SCFAs such as acetate, propionate, and butyrate, produced by the gut microbiota, can regulate the expression of tight junction proteins in brain endothelial cells, thereby enhancing BBB integrity (Fock and Parnova, 2023). On the other hand, the gut microbiota can maintain BBB function by modulating the immune system and reducing inflammation-induced damage to the BBB (Macpherson et al., 2023). In experimental autoimmune encephalomyelitis (EAE) mouse models, dysbiosis in the gut microbiota leads to increased BBB permeability, allowing immune cells and inflammatory factors to more easily enter the brain, exacerbating neuroinflammation and tissue damage. However, when EAE mice were treated with probiotics, the gut microbiota was regulated, SCFA production increased, the expression of tight junction proteins in the BBB returned to normal, permeability decreased, and neuroinflammation was alleviated (Zhang H. et al., 2022). This indicates that the gut microbiota plays a significant role in maintaining BBB integrity, and its imbalance may lead to impaired BBB function and contribute to the development of CNS diseases.

#### 3.5.2 The interaction between microbiota metabolites and the blood–brain barrier

Apart from short-chain fatty acids, other metabolites produced by the gut microbiota can also influence the function of the BBB. For instance, neurotransmitters such as dopamine and norepinephrine, although present in relatively low concentrations in the gut, can enter the brain via the bloodstream and modulate BBB function (Belousova et al., 2023; Mészáros et al., 2025). Additionally, the gut microbiota generates small molecule metabolites like indole and its derivatives, which possess anti-inflammatory and antioxidant properties and can protect the BBB from damage (Feng et al., 2023). Studies have shown that in patients with Parkinson’s disease (PD), alterations in the gut microbiota lead to reduced production of indole and its derivatives, resulting in impaired BBB function and facilitating the entry of neurotoxins into the brain, thereby exacerbating neuronal damage and death (Munoz-Pinto et al., 2024). By supplementing with probiotics rich in indole-producing bacteria or administering indole-like compounds, it is possible to improve BBB function and alleviate PD symptoms (Kim et al., 2023). This further underscores the crucial role of gut microbiota metabolites in maintaining BBB integrity and preventing CNS diseases.

### 3.6 The relationship between microbiota and neurodevelopment

#### 3.6.1 The impact of early microbiota colonization on neurodevelopment

**Figure 1:**
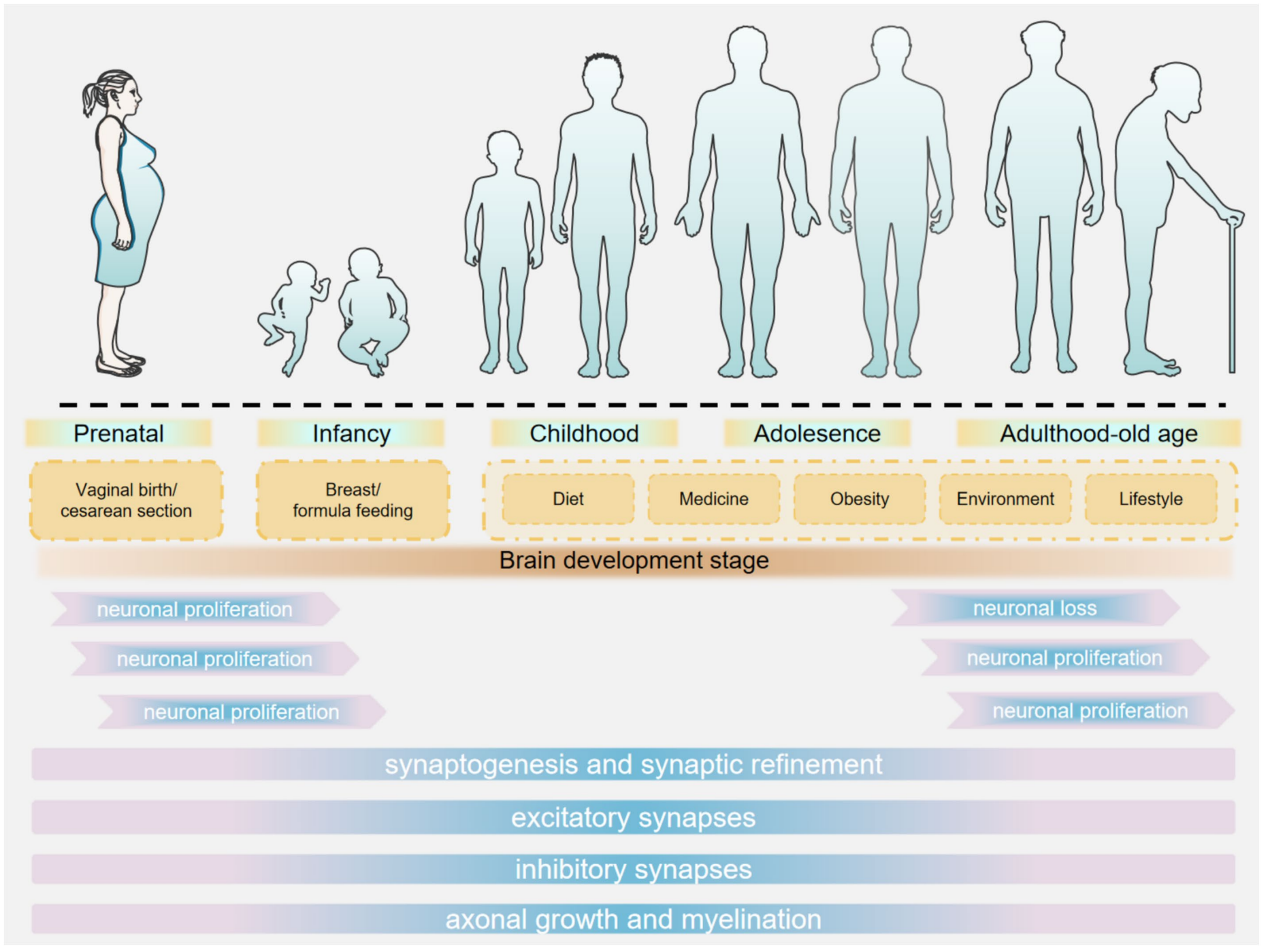
Stages of brain development.

During infancy and early childhood, the early colonization of the gut microbiota plays a crucial role in neurodevelopment (Frerichs et al., 2024). After birth, the gut microbiota rapidly establishes itself, influenced by factors such as mode of delivery, feeding method, and environment (Pantazi et al., 2023). Infants delivered vaginally are exposed to maternal vaginal and gut microbiota during birth, whereas those born via cesarean section are predominantly exposed to hospital environmental microbiota. This differential exposure contributes to variations in the composition of early-life intestinal microbiota and is associated with an increased risk of neurodevelopmental disorders (Bobba et al., 2023). Studies have demonstrated that vaginal microbiota transplantation (VMT) can expedite the maturation of intestinal microbiota in cesarean-born infants and enhance their neurodevelopment through the upregulation of specific intestinal metabolites and metabolic pathways (Zhou et al., 2023b). Breastfed infants have higher levels of beneficial bacteria such as *Bifidobacterium* in their gut, whereas formula-fed infants exhibit different diversity and composition of gut microbiota (Xu et al., 2024). Guo et al. (2023) demonstrated that breastfeeding, compared to formula feeding or mixed feeding, is advantageous for shaping the gut microbiome structure in preterm infants, enhancing language and cognitive scores, and promoting neurodevelopment. Various factors in the living environment can influence the establishment of the gut microbiota. For example, the diversity and abundance of environmental microorganisms affect the microbial exposure of infants, which in turn shapes the composition of their gut microbiota. Factors such as urban–rural differences, household hygiene practices, and pet ownership may all contribute to variations in the microbial communities infants are exposed to Wang M. et al. (2024). Rothenberg et al. (2021) conducted a cross-sectional analysis of the relationship between neurodevelopment and gut microbiota composition in 3-year-old children from rural China. They found that children who consumed fish within the past 24 h exhibited higher microbial abundance compared to those who did not, and this was positively correlated with both the intelligence development index and the psychomotor development index (Figure 1).

#### 3.6.2 The impact of the microbiota on neural plasticity

Neural plasticity refers to the nervous system’s ability to adapt to environmental changes during development and in adulthood (Johnson and Cohen, 2023). The gut microbiota can influence neural plasticity through multiple pathways, including regulating neurotransmitter levels, modulating neurotrophic factor expression, and controlling neuroinflammation (Damiani et al., 2023). For instance, SCFAs produced by the gut microbiota can regulate the expression of brain-derived neurotrophic factor (BDNF), a crucial neurotrophic factor that plays a significant role in neuronal survival, differentiation, and synaptic plasticity (Fang et al., 2023). In patients with depression, impaired neural plasticity is a key pathophysiological mechanism. Studies have shown that dysbiosis in the gut microbiota of depressed patients leads to reduced BDNF expression and decreased neural plasticity (Matin and Dadkhah, 2024). By regulating the gut microbiota and increasing SCFA production, BDNF expression can be enhanced, promoting the recovery of neural plasticity and thereby improving depressive symptoms (Palepu et al., 2024). Additionally, the gut microbiota plays a critical role in learning and memory processes. Experimental studies have demonstrated that germ-free mice perform significantly worse in learning and memory tasks compared to conventionally raised mice. However, when germ-free mice receive microbiota transplantation, their learning and memory abilities are significantly improved (Hoban et al., 2018). This indicates that the impact of the gut microbiota on neural plasticity is essential for maintaining and enhancing cognitive functions.

### 3.7 Other potential mechanisms by which the microbiota affects central nervous system diseases

#### 3.7.1 The interaction between the microbiota and the neuroendocrine system

The neuroendocrine system is a critical regulator of physiological functions in the body and is closely intertwined with the CNS (Knezevic et al., 2023). The gut microbiota can influence the occurrence and progression of CNS diseases through its interaction with the neuroendocrine system (Awe et al., 2024). For instance, the gut microbiota can modulate the function of the HPAA, a key stress regulatory system that secretes cortisol and other hormones in response to stress (Rusch et al., 2023). Under chronic stress conditions, dysbiosis in the gut microbiota can lead to HPAA dysfunction and abnormal increases in cortisol secretion (Zinkow et al., 2024). Prolonged high levels of cortisol can have detrimental effects on the CNS, causing neuronal damage, neurotransmitter imbalances, and neuroinflammation, thereby increasing the risk of mental disorders such as depression and anxiety (Dienes et al., 2013). By regulating the gut microbiota and restoring normal HPAA function, it is possible to mitigate the adverse effects of chronic stress on the CNS and improve the prevention and treatment of related mental disorders (Hao et al., 2023).

#### 3.7.2 Neuroactive substances produced by the microbiota

In addition to participating in the synthesis and regulation of neurotransmitters, the gut microbiota can directly produce various neuroactive substances, such as neuropeptides and neurotransmitter analogs (Hamamah and Covasa, 2022). These substances can enter the CNS via the bloodstream or neural pathways, influencing neuronal function and signal transduction. For instance, the species of *Bacteroides* containing *Bacteroides thetaiotaomicron*, *Bacteroides uniformis*, and *Bacteroides salyersiae* can produce gamma-aminobutyric acid analogs, which can bind to GABAR in the brain and regulate neural excitability (Conn et al., 2024). Moreover, the gut microbiota can generate neuroactive substances with antioxidant and anti-inflammatory properties, such as indole-3-propionic acid (IPA). These substances protect neurons from oxidative stress and inflammatory damage, thereby maintaining CNS homeostasis (Wang T. et al., 2023). Studies have shown that the levels of neuroactive substances produced by the gut microbiota are altered in neurodegenerative diseases such as PD and AD, which may be closely associated with the pathogenesis and progression of these diseases (Chen et al., 2022; Li L. et al., 2024). By modulating the gut microbiota and enhancing the production of beneficial neuroactive substances, new therapeutic strategies for neurodegenerative diseases may be developed (Yılmaz and Gökmen, 2020).

**Figure 2:**
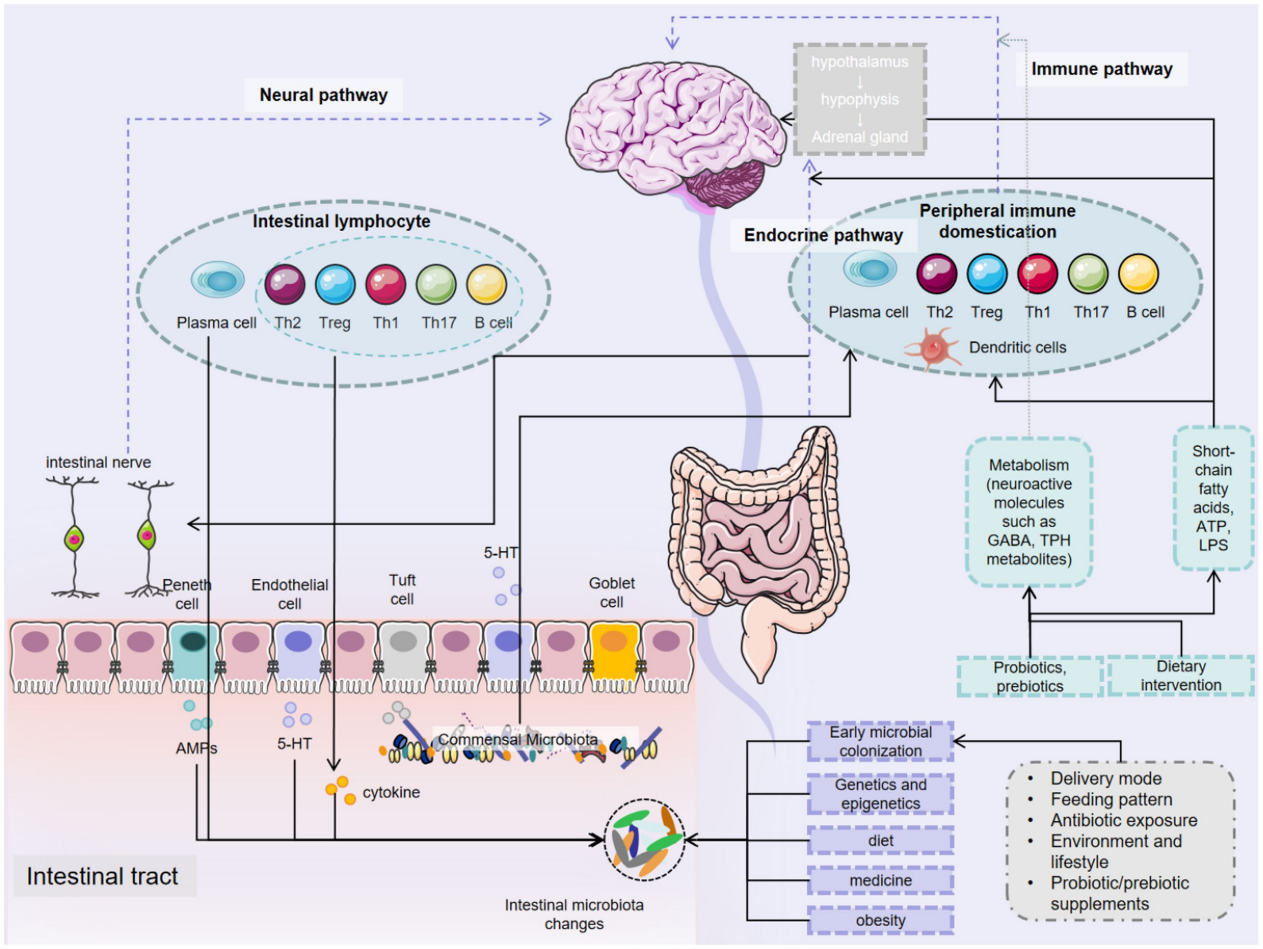
Gut microbiota promotes brain health.

In summary, the gut microbiota exerts profound influences on the occurrence, development, and treatment of CNS diseases through a variety of intricate mechanisms. These mechanisms include the synthesis and regulation of neurotransmitters, immune modulation, maintenance of the BBB, effects on neural development, interactions with neurotransmitter receptors, and associations with the neuroendocrine system. The gut microbiota plays an indispensable role in both the physiological and pathological processes of the CNS (Figure 2). In-depth research into how the microbiota affects CNS diseases not only enhances our understanding of their pathogenesis but also opens up broad prospects for developing novel diagnostic and therapeutic strategies based on microbiota manipulation.

## 4 The mechanism of MGB axis in central nervous system diseases

### 4.1 Multiple sclerosis

Multiple sclerosis (MS) is a common autoimmune disease of the CNS, characterized by the immune system erroneously attacking the myelin sheath, leading to impaired nerve conduction and a range of neurological symptoms such as limb weakness, vision loss, and balance disorders (Yamout et al., 2024). Recent studies have increasingly shown that MS patients exhibit significant alterations in gut microbiota composition, marked by a reduction in beneficial bacteria and a relative increase in pathogenic bacteria (Nouri et al., 2014; Altieri et al., 2023). This dysbiosis disrupts the normal differentiation and function of immune cells, particularly leading to excessive activation of Th1 and Th17 cells. Th1 cells primarily secrete pro-inflammatory cytokines such as interferon-γ (IFN-γ), while Th17 cells produce interleukin-17 (IL-17). The overproduction of these inflammatory cytokines can penetrate the blood–brain barrier, triggering neuroinflammation within the CNS, which exacerbates demyelination and contributes to the progression of MS (van den Bosch et al., 2023). Recent studies have demonstrated that an enhanced abundance of beneficial microorganisms is associated with a reduction in neuroinflammation in both MS and experimental autoimmune encephalomyelitis (EAE), a widely used mouse model for MS (Sell et al., 2022; Doyle et al., 2023). Additionally, certain microbial antigens share structural similarities with proteins in the myelin sheath, potentially inducing molecular mimicry reactions. When the immune system targets these microorganisms, it may mistakenly recognize and attack myelin due to structural similarities, causing damage to the myelin sheath (Fujinami and Oldstone, 1985). For example, the U24 protein of HHV-6 exhibits significant amino acid sequence homology with myelin basic protein (MBP) in the central nervous system’s myelin sheath. Studies have demonstrated that specific regions of the HHV-6 U24 protein, such as residues 10–20, share sequence similarities with immunodominant epitopes of MBP, including MBP85-99 (Tejada-Simon et al., 2003). This molecular mimicry can result in the immune system’s T cells or antibodies mistakenly recognizing and attacking MBP within the myelin sheath during viral clearance, leading to demyelinating lesions in the central nervous system. In the copper acetate-induced demyelination model, research demonstrated that the β-diversity of the gut microbiota in mice was altered. Furthermore, the relative abundance of certain species, such as *Eisenbergiella* and *Faecalibaculum*, exhibited a positive correlation with both the extent of demyelination in the central nervous system (CNS) and the degree of microglial activation (Wang et al., 2022). Additionally, following subdiaphragmatic vagotomy in mice treated with copper acetate, demyelination in the corpus callosum and microglial activation were attenuated. Moreover, the abnormal β-diversity of the gut microbiota was partially restored, as evidenced by an increase in the relative abundance of *Lactobacillus* and *Turicibacter* (Wang X. et al., 2023). These findings indicate that the vagus nerve, as a critical component of the gut-brain axis, plays a pivotal role in the pathological mechanism underlying copper acetate-induced CNS demyelination.

### 4.2 Autism spectrum disorder

Autism Spectrum Disorder (ASD) is a group of neurodevelopmental disorders characterized by social impairments, delayed language development, repetitive and stereotyped behaviors, and restricted interests (Hirota and King, 2023). Recent studies have increasingly highlighted the significant role of gut microbiota in both the pathogenesis and therapeutic intervention of ASD (Korteniemi et al., 2023). The reduction of Bacteroidota/Bacillota ratio (basonym Bacteroidetes/Firmicutes), the abundance of Bacteroidota (basonym Bacteroidetes) and other imbalances in the gut of ASD patients may competitively consume tryptophan, leading to a decrease in serotonin synthesis (Settanni et al., 2021; Wegiel et al., 2024). The resulting decrease in serotonin levels can disrupt neural signaling in the brain, thereby affecting social behavior and emotional regulation, which may play a critical role in the onset and progression of ASD (Lee et al., 2022). Additionally, research has shown that ASD patients exhibit a reduction in beneficial bacteria such as *Bifidobacterium* and *Lactobacillus*, while harmful bacteria like *Clostridium* are relatively increased. This dysbiosis can stimulate the immune system to produce excessive inflammatory factors, including tumor necrosis factor-α (TNF-α) and interleukin-6 (IL-6). These inflammatory mediators can enter the brain via the bloodstream or neural pathways, triggering neuroinflammation, impairing normal neuronal development and function, and disrupting the formation and signal transmission of neural circuits, ultimately contributing to the manifestation of ASD-related symptoms (Hughes et al., 2018).

A comparative study between children with ASD and typically developing children revealed significant alterations in the diversity, stability, and composition of the intestinal microbiota in ASD children. Specifically, the abundance of *Bacteroides*, *Parabacteroides*, *Clostridium*, *Faecalibacterium*, and *Clostridiaceae* increased, while that of *Bifidobacterium*, *Coprococcus*, and *Akkermansia* decreased (Xu et al., 2019; Iglesias-Vázquez et al., 2020). Concurrently, ASD children exhibited elevated levels of inflammatory factors in their blood and increased intestinal permeability, consistent with immune dysregulation and impaired intestinal barrier function resulting from microbial imbalance. These findings further substantiate the association between gut microbiota and ASD. In animal models, transplanting the gut microbiota from ASD patients into germ-free mice induced ASD-like behavioral characteristics, including reduced social interactions and increased repetitive behaviors. However, probiotic intervention in these mice led to a normalization of the gut microbiota and an improvement in ASD-like behavioral symptoms. This evidence suggests that the gut microbiota plays a crucial role in the pathogenesis of ASD and that modulating the microbiota may offer therapeutic potential for improving ASD symptoms (Sharon et al., 2019; Andreo-Martínez et al., 2022).

### 4.3 Alzheimer’s disease

Alzheimer’s disease (AD) is a degenerative neurological disorder that accounts for 60–80% of all dementia cases in the elderly. Currently, it is estimated that there are over 55 million dementia patients worldwide, and this number may reach 150 million by 2050, imposing a significant societal burden (Janoutová et al., 2021). The most common manifestations in AD patients include cognitive impairment and neuropsychiatric symptoms. The primary pathological mechanisms involve abnormal deposition of amyloid beta-protein (Aβ) and excessive phosphorylation of tau protein, leading to neurofibrillary tangles and oxidative stress, among other factors (Chen and Yu, 2023; Sigurdsson, 2024). Emerging evidence has established a link between the gut microbiota and AD pathology. Mice fed a fiber-deficient diet showed a significant reduction in Bacteroidota (basonym Bacteroidetes) and an increase in Pseudomonadota (basonym Proteobacteria), which damaged the intestinal barrier and decreased the production of SCFAs, resulting in cognitive decline (Shi et al., 2021). In animal models, AD mice exhibited elevated levels of Bacteroidota (basonym Bacteroidetes) and a loss of protective effects from *Bifidobacterium*, along with increased expression of inflammasomes and interleukin-1β (IL-1β) in the brain, indicating that gut microbiota can trigger AD pathology through neuroinflammation (Shukla et al., 2021). In a recent study, a specific strain of *Faecalibacterium prausnitzii* isolated from healthy participants was found to alleviate cognitive deficits in a mouse model of cerebral amyloidosis (Ueda et al., 2021). Similarly, in the 5xFAD mouse model of AD, dietary supplementation with SCFAs modulated by gut microbiota-derived mannan oligosaccharides effectively suppressed neuroinflammation and mitigated cognitive dysfunction (Liu et al., 2021). Furthermore, in a phase 2 randomized controlled trial, sodium oligomannate, an oligosaccharide compound derived from seaweed, demonstrated its efficacy in improving cognitive outcomes in AD patients (Wang et al., 2020). Collectively, AD patients exhibiting intestinal microbiota dysbiosis are more likely to experience impaired overall cognitive and memory functions. The underlying mechanisms may involve the promotion of Aβ deposition, neuroinflammation, and oxidative stress, among others. These findings suggest that alterations in the intestinal microbiota could represent a potential pathological contributor to AD.

### 4.4 Parkinson’s disease

Parkinson’s disease (PD) is a multifactorial clinical syndrome and the second most common neurodegenerative disorder globally, contributing significantly to neurological dysfunction (Chu et al., 2024). The pathogenesis of PD is complex and not yet fully understood, but known mechanisms include neuroinflammation, oxidative stress, and mitochondrial dysfunction (Morris et al., 2024). In 2021, the global prevalence of PD was estimated at approximately 11.9 million patients. Given the ongoing trend of population aging, it is projected that by 2050, the number of PD patients worldwide will reach 25.2 million, representing an increase of 112% compared to 2021 (Su et al., 2025). PD patients frequently exhibit gastrointestinal symptoms that precede motor symptoms. The neuropathology of PD is closely linked to the enteric nervous system, and changes in gut microbiota are considered potential environmental triggers of PD pathology (Kalyanaraman et al., 2024).

The precise mechanisms connecting the neurobiological signals of the gut microbiota to the CNS remain unclear, with various hypotheses proposed. Among these, the widely recognized Braak hypothesis posits that disturbances in the intestinal system can alter the composition of the gut microbiota, promoting the production of intestinal toxins. These toxins induce the misfolding of α-synuclein, leading to CNS inflammation, activation of brain microglia, and damage to dopaminergic neurons, which further results in GBA dysfunction. Misfolded α-synuclein can then propagate like prions, traveling through the vagus nerve to the lower brainstem and eventually reaching the midbrain, contributing to the development of PD (Ochoa-Repáraz et al., 2020; Rea et al., 2020; Kamienieva et al., 2021). Recent studies have identified 84 out of 257 isolated gut microbial taxa as being associated with PD, including *Prevotellaceae*, *Bacteroides*, *Faecalibacterium*, *Shigella*, *Streptococcus*, *Desulfovibrio*, and *Enterococcus* (Wallen et al., 2022). The metabolic products of these microbes play a crucial role in PD pathogenesis. Disruptions in the gut microbiota lead to reduced levels of *Prevotella* and *Ruminococcus*, resulting in decreased mucus protein secretion. As mucus proteins serve as nutrients for other bacteria, their degradation increases intestinal permeability, allowing pathogenic microbial metabolites to enter the systemic circulation (Salim et al., 2023). Probiotics can reverse alterations in gut microbiota composition, restore gastrointestinal function, reduce intestinal leakage and enteric nervous system inflammation, exert antioxidant effects, improve mitochondrial function, and enhance energy metabolism in the brain and muscles, thereby preventing motor deficits caused by muscle atrophy and reducing dopaminergic neuron loss (Hanscom et al., 2021). Metabolic products such as hydrogen sulfide from *Prevotella* exhibit neuroprotective properties. Other key metabolites include butyrate, which degrades α-synuclein via the autophagy pathway involving Atg5, inhibits demyelination, enhances myelin regeneration, promotes oligodendrocyte differentiation, and protects the nervous system. Butyrate and propionate also regulate tyrosine hydroxylase synthesis by modulating the tyrosine hydroxylase gene, thereby reducing dopamine secretion in PD patients. Acetic acid serves as a substrate for the synthesis of butyrate and propionate by gut microbiota (Salim et al., 2023).

### 4.5 Amyotrophic lateral sclerosis

Amyotrophic lateral sclerosis (ALS) is a fatal neurodegenerative disease characterized by rapid progression and an unknown etiology. Its primary clinical manifestations include symptoms and signs of upper and lower motor neuron damage, such as muscle atrophy, weakness, and pyramidal tract signs (Riva et al., 2024). The disease ultimately progresses to the point where patients die from complications such as swallowing difficulties or respiratory failure. In addition to motor symptoms, ALS patients may also experience non-motor symptoms, including cognitive impairment, fatigue, pain, and depression (Shojaie et al., 2023). Research has shown that the median survival period from symptom onset to death for ALS patients is approximately 2–5 years (de Falcão Campos et al., 2022). Currently, there are no effective treatments for ALS. Only two drugs, riluzole and edaravone, have been proven to temporarily slow disease progression, but they do not alter the ultimate outcome for patients (Ilieva et al., 2023). In recent years, significant progress has been made in understanding the pathogenesis of ALS. More than 20 gene mutations, such as SOD1, C9orf72, TARDBP, and FUS, have been identified as potential causes of most familial and some sporadic cases of ALS. Additionally, the role of environmental factors, including gut microbiota dysbiosis, and the combined effects of environment and genetics in ALS pathogenesis have gradually gained recognition (Niccolai et al., 2024). Advances in high-throughput detection technologies have facilitated substantial progress in characterizing the composition of the gut microbiota. Research on the correlation between ALS and the gut microbiota has evolved from descriptive analyses of microbial composition to investigations into the causal relationship between microbiota imbalance and ALS pathogenesis.

Currently, it is widely recognized by scholars both domestically and internationally that the gut-brain axis plays a crucial role in the pathogenesis of amyotrophic lateral sclerosis (ALS). Studies have shown that a higher Bacteroidota/Bacillota ratio (basonym Bacteroidetes/Firmicutes) in ALS patients is associated with an increased risk of mortality, and the diversity of the gut microbiota is also linked to patient survival outcomes (Chen et al., 2024). Research has demonstrated that intestinal microbial metabolites, such as nicotinamide (the amide form of vitamin B3), are involved in energy metabolism. In SOD1G93A mice, decreased levels of nicotinamide in serum and cerebrospinal fluid corresponded with reduced numbers of *Akkermansia muciniphila* in the gut. Transplanting *Akkermansia muciniphila* into these mice improved their survival period (Blacher et al., 2019), further confirming that changes in microbial metabolite levels can influence ALS progression. Studies on SOD1G93A mouse models revealed that before symptom onset at 8–9 weeks of age, there was a reduction in gut microbial abundance, leading to decreased production of SCFAs such as acetic acid, propionic acid, and butyric acid through microbial fermentation. This decrease in SCFAs was associated with increased intestinal permeability, and supplementation with butyric acid and propionic acid restored intestinal barrier integrity (Niccolai et al., 2024). Increased intestinal permeability exacerbates the release of signaling factors, particularly inflammatory cytokines, which enter the bloodstream and trigger systemic inflammation, thereby activating the neuroimmune system. Chronic inflammation in the CNS contributes to the progression of neurodegenerative diseases, including PD, AD, and ALS. Animal studies have shown that compared to wild-type mice, G93A mice exhibit distinct differences in fecal microbiota composition, with increased numbers of Paneth cells, which sense microorganisms and secrete antimicrobial peptides. Dysbiosis of the gut microbiota and damage to tight junctions occur before the onset of ALS symptoms, accompanied by increased intestinal permeability (Xin et al., 2024).

### 4.6 Huntington’s disease

Huntington’s disease (HD), also known as Huntington’s chorea, is an autosomal dominant neurodegenerative disorder characterized by the mutation of the huntingtin gene on chromosome 4. This mutation leads to the production of a mutant huntingtin protein that gradually accumulates within cells, forming large molecular aggregates. These aggregates accumulate in the brain, disrupting neuronal function (Saade and Mestre, 2024). Typically, HD manifests in midlife with choreiform movements. As the disease progresses, patients progressively lose the ability to speak, move, think, and swallow. The disease course generally spans 10 to 20 years, ultimately leading to death (Oosterloo et al., 2024). Although HD is primarily a genetic disorder, the onset and severity of symptoms are influenced by various environmental factors, including diet, physical activity, and stress (Tong H. et al., 2024). Emerging evidence suggests that the gut microbiota may also play a role in the pathogenesis and progression of HD (Sharma et al., 2023).

Two independent studies on mouse models and one clinical study have demonstrated that patients with HD exhibit intestinal dysbiosis (Radulescu et al., 2020; Wasser et al., 2020). Research has shown that in transgenic mouse lines modeling HD, intestinal homeostasis is disrupted, and the composition of the gut microbiota is altered, revealing instability in the HD gut microbiome during the pre-motor stage, which can have serious consequences for host health (Gubert et al., 2022). Before significant cognitive and motor impairments manifest, functional disturbances in the gut microbiome of HD patients suggest an important role of the intestine in regulating HD pathogenesis. This may occur through specific changes in plasma metabolites that mediate gut-brain signaling, such as butyrate and SCFAs (Kong et al., 2021). Clinical studies comparing the gut microbiota of HD gene expansion carriers with healthy controls have found a significant reduction in both the quantity and diversity of gut microbiota in HD carriers (Wasser et al., 2020). In summary, the gut microbiota can influence HD progression by affecting brain function through their metabolic products, although the specific mechanisms remain unclear and require further investigation. Current findings highlight the importance of gut biomarkers in HD and suggest potential targets for future therapeutic interventions aimed at modulating the gut microbiota in HD patients.

## 5 Factors affecting the microbiota-gut-brain axis

### 5.1 Genetics and epigenetics

The relationship between host genetics and microbiota composition is an important yet underexplored research area, particularly in the context of brain health. Genetics, through gene coding, determines the fundamental characteristics and functions of an organism and has a profound impact on the construction and regulation of the gut-brain axis (Wilde et al., 2024). From the perspective of gut microbiota, the host’s genetic background can influence both the composition and abundance of the intestinal microbiota (Lee et al., 2023). Studies have shown that individuals with different genetic backgrounds exhibit significant differences in the types and quantities of microorganisms in their intestines (Cammann et al., 2023). For instance, certain gene polymorphisms are associated with the enrichment or depletion of specific bacterial taxa in the gut. Specific genetic variations may alter the intestinal microenvironment, such as the composition of the mucus layer and immune cell secretion patterns, thereby affecting microbial colonization and survival (Parizadeh and Arrieta, 2023). This genetic shaping of the gut microbiota, in turn, influences the signaling within the gut-brain axis. The gut microbiota communicates with the brain via metabolic products such as SCFAs and neurotransmitters, and the ability of the gut microbiota to produce these metabolites varies across different genetic backgrounds, ultimately impacting neural function and behavioral outcomes (Sugiyama and Murayama, 2023). From the perspective of the nervous system, genetic factors determine the structure and function of neurotransmitter systems, which are crucial for gut-brain axis signaling. For example, serotonin (5-hydroxytryptamine), an important neurotransmitter, plays a key role in regulating mood, cognition, and gastrointestinal function. Mutations or polymorphisms in genes encoding enzymes involved in serotonin synthesis, transporters, and receptors can alter serotonin levels and signaling efficiency, thereby affecting the bidirectional regulation of the gut-brain axis (Kang et al., 2024). Studies have found that certain serotonin-related gene variants are associated with an increased risk of mental disorders such as anxiety and depression, as well as gastrointestinal conditions like irritable bowel syndrome (IBS), underscoring the importance of genetic factors in gut-brain axis-related diseases (Licht et al., 2020; Bousman et al., 2023). Epigenetics regulates gene expression without altering the DNA sequence through mechanisms such as DNA methylation, histone modification, and non-coding RNA regulation, playing a significant role in the gut-brain axis (Jeong et al., 2023). In the intestine, epigenetic modifications can influence the function of intestinal epithelial cells and the differentiation of immune cells. For example, changes in DNA methylation patterns can regulate the expression of genes related to intestinal barrier integrity, affecting mucosal permeability (Zhao et al., 2024). When the intestinal barrier is compromised, bacteria and their metabolic products can more easily enter the bloodstream, potentially triggering neuroinflammatory responses in the brain. Studies have shown that patients with inflammatory bowel disease (IBD) exhibit specific DNA methylation abnormalities in intestinal tissues, which are linked to disease progression and gut-brain axis dysfunction (Xu et al., 2023). In the brain, epigenetic modifications also impact neuronal function and neural circuit formation. Histone modifications can regulate the expression of genes involved in neural plasticity, neurotransmitter synthesis, and metabolism. In neuropsychiatric disorders such as depression and autism, abnormal epigenetic marks in the brain may interfere with normal gut-brain axis signaling, leading to intestinal dysfunction and behavioral abnormalities (Mouat and LaSalle, 2022; Zhu et al., 2023).

### 5.2 Mode of delivery at birth

With the improvement of healthcare systems and increased disposable income, more mothers are opting for cesarean section (Cesarean Delivery, CS) as their preferred mode of delivery. Studies have shown that infants delivered by cesarean section, due to bypassing the birth canal, miss out on exposure to beneficial maternal vaginal and intestinal bacteria, leading to delayed and altered early intestinal microbiota colonization compared to vaginally delivered infants (Inchingolo et al., 2024). Cesarean section infants exhibit a higher proportion of hospital-acquired microorganisms such as *Staphylococcus* and *Corynebacterium* in their intestines, while the colonization of beneficial bacteria like *Bifidobacterium* and *Lactobacillus* is delayed and less abundant (Zhou et al., 2023b). This difference in microbiota structure can persist for an extended period, potentially impacting the normal development of the immune system and neurodevelopment in infants and young children. For example, insufficient early exposure to diverse microbiota may cause the immune system to overreact or respond abnormally to subsequent antigenic stimuli, leading to immune dysregulation (Lai et al., 2024). Chronic immune imbalance can result in persistent inflammation, with inflammatory factors potentially crossing the blood–brain barrier and causing damage to neural cells, thereby affecting neurodevelopment and increasing the risk of brain-related disorders (Lin et al., 2023). Studies have found that children born via cesarean section have a higher incidence of allergic and autoimmune diseases, which may share common pathophysiological mechanisms with neurodevelopmental disorders (Moore et al., 2023; Liu X. et al., 2024). Moreover, changes in the intestinal microbiota of cesarean section infants can influence the synthesis and metabolism of neurotransmitters (Joe et al., 2022). As previously discussed, the gut microbiota plays a crucial role in the synthesis of neurotransmitters such as serotonin. Abnormalities in the gut microbiota of cesarean section infants may lead to insufficient serotonin synthesis or metabolic disturbances, thereby disrupting neural signaling in the brain and altering the expression of genes involved in neurodevelopment. This can make infants and young children more susceptible to neurodevelopmental issues and increase the risk of brain-related diseases (Bobba et al., 2023).

### 5.3 Diet

Diet is a critical factor in shaping the structure and function of the gut microbiota (Fan et al., 2023). Dietary fiber, a type of carbohydrate that cannot be directly broken down by human digestive enzymes, serves as an essential energy source for beneficial gut bacteria. Beneficial bacteria such as *Bifidobacterium* and *Lactobacillus* can ferment dietary fiber to produce SCFAs like acetate, propionate, and butyrate (Holmberg et al., 2024). These SCFAs not only provide energy for intestinal epithelial cells and maintain the integrity of the intestinal mucosa but also influence brain neural activity through the gut-brain axis (Mann et al., 2024). Studies have shown that SCFAs can regulate neurotransmitter levels in the brain, such as increasing serotonin synthesis, thereby enhancing mood and cognitive function (Zhong et al., 2023). In contrast, a high-sugar diet, particularly one rich in simple sugars like sucrose and fructose, can lead to dysbiosis of the gut microbiota (Gao et al., 2024). Excessive monosaccharides can be rapidly metabolized by harmful bacteria in the gut, promoting their rapid proliferation, such as *Escherichia coli* and *Clostridioides difficile* (basonym *Clostridium difficile*). The overgrowth of these pathogenic bacteria may suppress the growth of beneficial bacteria, disrupt the balance of the gut microbiota, and trigger intestinal inflammation (Zhang et al., 2025). The content and type of fat in the diet also significantly impact the gut microbiota and, consequently, brain health. A high-saturated-fat diet can reduce gut microbiota diversity, decrease the abundance of beneficial bacteria, and increase the presence of harmful bacteria. Research has found that long-term consumption of a high-fat diet (60% fat content) leads to a decline in protective bacterial genera such as *Lactobacillus* spp., *Bifidobacterium* spp., and *Bacteroides-Prevotella* spp., while the abundance of sulfate-reducing bacteria that produce hydrogen sulfide and LPS, which damage the intestinal barrier, significantly increases (Daniel et al., 2014). This diet alters the diversity and abundance of gut microbiota in mice, leading to a reduction in *Oscillospiraceae* and an increase in *Rikenellaceae*, both of which are associated with intestinal inflammation and metabolic diseases. Intestinal inflammation is linked to neuroinflammation in the brain, potentially resulting in cognitive decline and mood disorders (Cavaliere and Traina, 2023). Conversely, unsaturated fatty acids, especially omega-3 polyunsaturated fatty acids (PUFAs), exhibit anti-inflammatory and gut microbiota-regulating properties (Brosolo et al., 2024). Omega-3 PUFAs can modulate the immune response in the gut, inhibit the growth of harmful bacteria, and promote the colonization of beneficial bacteria, such as increasing the numbers of *Bifidobacterium* and *Lactobacillus*, thereby improving the composition of the gut microbiota (Bascuñán et al., 2025). Additionally, omega-3 PUFAs can act directly on the brain, participating in the synthesis of neural cell membranes, enhancing neural cell activity, and improving cognitive function and mood (Zailani et al., 2024).

### 5.4 Drugs

Among the various factors influencing the gut-brain axis, drugs play a significant and multifaceted role. Their mechanisms of action on the gut-brain axis are complex and diverse, affecting the intestinal microbiota, intestinal barrier function, and the metabolism and signaling of neurotransmitters, either directly or indirectly.

Antibiotics are a common class of drugs that significantly impact the gut-brain axis. Extensive research has shown that antibiotic use can lead to dysbiosis in the intestinal microbiota (Fishbein et al., 2023). For instance, a study on mice found that administration of broad-spectrum antibiotics resulted in a marked reduction in beneficial bacteria such as *Bifidobacterium* and *Lactobacillus*, while promoting the proliferation of antibiotic-resistant bacteria (Vicentini et al., 2021). This alteration in microbiota composition further disrupted the metabolic functions of the gut microbiota, leading to a notable decrease in the production of SCFAs. Psychotropic drugs also have a close connection with the gut-brain axis. Selective serotonin reuptake inhibitors (SSRIs), widely used for treating depression and anxiety, not only regulate serotonin levels in the brain but also influence the intestinal microbiota (Brushett et al., 2023). Clinical studies have shown that patients taking SSRIs experience changes in the diversity and composition of their gut microbiota, with an increase in the abundance of some beneficial bacteria and a decrease in certain harmful ones (Jiang Y. et al., 2024). This effect may be due to the fact that serotonin, an important neurotransmitter in the brain, is also abundant in the gut and plays a crucial role in processes such as peristalsis, secretion, and immune regulation. The regulatory effect of SSRIs on serotonin indirectly influences the intestinal microenvironment, and changes in the gut microbiota may feedback-regulate brain neural functions through the gut-brain axis, potentially affecting the therapeutic efficacy of these drugs (Diviccaro et al., 2022). Proton pump inhibitors (PPIs), commonly used to treat acid-related gastrointestinal diseases, have garnered increasing attention for their impact on the gut-brain axis. PPIs alter the pH of the gastrointestinal tract by inhibiting gastric acid secretion, which in turn affects the composition and activity of the intestinal microbiota (Kiecka and Szczepanik, 2023). Studies have demonstrated that long-term PPI use can change the composition of the gut microbiota and lead to bacterial translocation from the oral cavity to the intestinal lumen (Shi et al., 2019; Jang et al., 2020). A cohort study on elderly individuals found an association between long-term PPI use and cognitive decline, which may be related to the effects of PPIs on the gut-brain axis (Northuis et al., 2023).

### 5.5 Obesity

Obesity, an increasingly prevalent metabolic disorder, has been shown to exert complex and extensive effects on the gut-brain axis. Obesity is closely associated with dysbiosis of the intestinal microbiota (Puljiz et al., 2023). Multiple studies have demonstrated that the structure of the intestinal microbiota in obese individuals differs significantly from that in individuals with normal weight. For instance, in a study involving monozygotic twins, the gut microbiota of obese and normal-weight individuals were analyzed. The results indicated that the abundance of *Bacteroides and Collinsella* was significantly lower in the obese group compared to their normal-weight counterparts (Yin et al., 2022). Additionally, obesity often induces a state of chronic low-grade inflammation, which can damage the intestinal mucosa and compromise the integrity of the intestinal barrier. Research has shown that in obese mice, the expression of tight junction proteins in the intestine decreases, leading to increased intestinal permeability and facilitating the translocation of harmful substances such as bacterial endotoxins into the bloodstream. Once these endotoxins enter the circulation, they activate the immune system, triggering a systemic inflammatory response (Cai et al., 2024). Inflammatory factors can cross the blood–brain barrier, entering the brain and inducing neuroinflammation.

### 5.6 Other factors

Other factors, including environmental factors (e.g., toxins, pesticides, infections, stressors) and lifestyle factors (e.g., exercise, sleep patterns), significantly influence the modulation of the gut microbiota-brain axis by shaping the interaction between the gut microbiota and the brain.

The rapid advancement of modern industry and agriculture has resulted in an increasing exploitation and utilization of heavy metals by humans. Heavy metals encompass both essential elements for life activities (such as iron, copper, and zinc) and toxic elements harmful to organisms (such as lead, cadmium, and arsenic) (Yu G. et al., 2024). The introduction of toxic metals into the complex intestinal ecosystem may disrupt normal intestinal functions. Alterations in the composition of gut microbiota can lead to the direct release of microbial by-products (including bacterial toxins and bacteria) into the bloodstream and/or modify the permeability of the intestinal barrier, thereby increasing the risk of acute infections and immune system dysregulation (Teffera et al., 2024). Studies have demonstrated that lead exposure significantly elevates the levels of Gram-negative bacteria in the intestine, which produce bacterial toxin LPS. LPS binds to the TLR4 receptor on immune cells, activating the LPS/TLR4 signaling pathway and triggering systemic inflammatory responses (Farhana and Khan, 2025). Arsenic, similar to lead, is a naturally occurring element in the environment, primarily present in soil and groundwater in organic and inorganic forms (Hu et al., 2025). Chen et al. (2025) found that arsenic exposure reduces the abundance of *Desulfovibrio fairfieldensis*, disrupts the intestinal microenvironment, and affects neural function. Cadmium, a heavy metal with extremely high ecological risks, can enter the body through ingestion by aquatic organisms or skin penetration, subsequently traveling via the bloodstream to various organs and tissues, causing irreversible severe damage (Wang et al., 2025). Research indicates that exposure to lead and cadmium decreases the relative abundance of Bacillota (basonym Firmicutes) and unidentified_Bacteria while increasing the relative abundance of Pseudomonadota (basonym Proteobacteria), Synergistota, and Bacteroidota (basonym Bacteroidetes). This alters the composition and structure of the gut microbiota, leading to neurotoxicity, liver and kidney damage, and metabolic disorders (Zhang S. et al., 2024). Atrazine, a herbicide that interferes with plant photosynthesis, has been shown to disrupt the endocrine systems of amphibians and mammals (Holliman et al., 2025). Studies reveal that atrazine exposure in frogs significantly reduces the diversity of their gut microbiota. At the highest tested dose (500 μg/L), the proportion of *Lactobacillus* and *Weissella* significantly increases. Additionally, frog jumping distance and time are significantly altered, potentially due to interference with motor neuron signal transmission (Zhao et al., 2021). Infections (e.g., viral or bacterial infections) may further impact MGBA signaling by activating the immune system or altering the balance of gut microbiota. For instance, *Helicobacter pylori*, a common gastric pathogen, can cross the intestinal barrier via transcellular pathways or indirectly enter the brain through the vagus nerve, increasing the density and surface area of Aβ plaques and influencing the pathological progression of Alzheimer’s disease (Xie et al., 2023). Psychological theories suggest a clear association between stress and the risk of mental disorders or diseases. Various stressors, particularly during childhood, can elevate the risk of mental disorders, including affective and anxiety disorders, by activating the HPAA, increasing cortisol levels, weakening neuronal plasticity processes, and altering the diversity and function of gut microbiota (Góralczyk-Bińkowska et al., 2022).

In addition, lifestyle factors such as exercise can modulate the composition of the gut microbiota and the production of metabolites, including LPS (Min et al., 2024). Notably, a study investigated the effects of high-intensity interval training (HIIT) and moderate-intensity continuous training (MICT) on the cognitive function of mice via the gut-brain axis, focusing on gut microbiota composition and LPS translocation. The findings suggested that HIIT may pose a potential risk by inducing “leaky gut” through alterations in microbiota associated with intestinal permeability. This leads to elevated levels of LPS in the blood and brain, activating M1 microglia in the brain, which subsequently reduces dendritic spine density and impairs cognitive function (Peng et al., 2024). Furthermore, maintaining a healthy sleep pattern contributes to the homeostasis of the gut microbiota and supports nervous system health (Zhang N. et al., 2024). Collectively, these factors interact to influence the balance of the MGBA, thereby profoundly affecting the onset and progression of central nervous system disorders.

## 6 Treatment approaches

### 6.1 Early intervention

In the early stages of life, the colonization and establishment of the intestinal microbiota are critical for the normal development of the immune system, neurodevelopment, and metabolic functions (Donald and Finlay, 2023). Interventions during this period can shape a healthy intestinal microbiota structure and lay the foundation for subsequent health. For instance, during infancy, when the intestinal microbiota is not yet stable, appropriate intervention measures such as breastfeeding, supplementation with specific probiotics or prebiotics, can promote the early colonization of beneficial bacteria and inhibit the growth of harmful bacteria (Ames et al., 2023). Breastfeeding not only provides infants with essential nutrients but also introduces a rich variety of probiotics from breast milk, such as *Bifidobacterium*, which helps establish a healthy intestinal microenvironment (Masi and Stewart, 2024). Moreover, the balance of the early intestinal microbiota aids in regulating immune system development and reducing the adverse effects of inflammatory responses on neurodevelopment. Studies have shown that the intestinal microbiota can influence neurotransmitter synthesis and neural circuit formation through the gut-brain axis. Early intervention in the intestinal microbiota can optimize these processes and positively impact the development of the central nervous system. For example, a study on premature infants found that supplementing them with probiotics (*Bifidobacterium* and *Lactobacillus*) in the first few weeks after birth led to a significant increase in the number of beneficial bacteria in their intestines, improved intestinal barrier function, and reduced levels of inflammatory factors (Baucells et al., 2023). More importantly, in subsequent neurodevelopmental assessments, the intervention group of premature infants performed better than the control group in cognitive, language, and motor development. This indicates that early probiotic supplementation has a positive effect on the neurodevelopment of premature infants, possibly by regulating the intestinal microbiota, improving the intestinal microenvironment, and mitigating inflammation-induced damage to neurodevelopment. In studies on children with autism spectrum disorder (ASD), it was found that early intervention in the intestinal microbiota of ASD children, such as supplementing with prebiotics or adjusting their diet, can help improve ASD symptoms (Zeng P. et al., 2024). This further underscores the importance of early intervention in the intestinal microbiota for the neurodevelopment and symptom improvement of ASD children.

### 6.2 Probiotics, prebiotics, synbiotics, and postbiotics

Probiotics are live microorganisms that colonize the human and animal intestines, maintaining the balance of the host’s intestinal microbiota and exerting beneficial effects on the host’s health (Mazziotta et al., 2023). Prebiotics are nondigestible dietary components that can be selectively fermented by the intestinal microbiota, promoting the growth and activity of beneficial bacteria in the gut, thereby conferring health benefits to the host (Vijaya et al., 2024). Probiotics and prebiotics have been demonstrated to exert beneficial effects in the prevention of Alzheimer’s disease, Parkinson’s disease, depression, autism spectrum disorder, and other neurological and mental health disorders (Ribera et al., 2024; Taghizadeh Ghassab et al., 2024; Zhang et al., 2024b; Ye et al., 2025). Multiple randomized controlled clinical trials have shown that the intake of multi-strain probiotics or their combination with prebiotic fibers can improve gastrointestinal symptoms in PD patients by modulating the microbiota-gut-brain axis, including reducing abdominal pain, bloating, and constipation (Parra et al., 2023; Ma et al., 2024; Yuan et al., 2024). A study demonstrated that prebiotics, particularly fructooligosaccharides (FOS) and galactooligosaccharides (GOS), enhance the gut microbiota of high-fat diet (HFD)-fed mice by increasing acetate-producing bacteria, such as *Bacteroides acidifaciens* and *Phocaeicola dorei* (basonym *Bacteroides dorei*), improving intestinal permeability, upregulating neurogenesis and synaptic plasticity-related markers (PSD, SAP 102, CREB-p, and BDNF), promoting brain acetate and GPR43 levels while reducing pro-inflammatory cytokines, and positively influencing neuronal proliferation and survival signaling in the hippocampus and prefrontal cortex (de Paiva et al., 2024). Synbiotics, which combine probiotics and prebiotics, not only enhance the physiological activity of probiotics but also selectively increase their abundance, inhibit pathogenic bacterial growth and metabolism, activate host immune responses, restore microecological balance, and exert beneficial effects (Lee et al., 2024). Deng et al. (2022) utilized a complementary synbiotic formulation containing inulin and a multispecies probiotic mixture, such as *Bacillus subtilis* var. natto, *Heyndrickxia coagulans* (basonym *Bacillus coagulans*), *Lacticaseibacillus casei* (basonym *Lactobacillus casei*), *Lactobacillus acidophilus*, *Bifidobacterium longum*, and *Bifidobacterium breve*, which improved memory deficits and hippocampal neurogenesis in Alzheimer’s disease (AD) mice while reducing Aβ42 and TNF-α expression. Tong Y. et al. (2024) reported that synbiotic treatment significantly enhanced gut microbial diversity, activated peroxisome proliferator-activated receptor (PPAR) signaling pathways, and markedly reduced neuroinflammation in AD mouse models. The International Scientific Association for Probiotics and Prebiotics updated the definition of postbiotics as “non-viable microbial cells and/or their components with health benefits to the host” (Salminen et al., 2021). Known postbiotics include intentionally inactivated microbial cells, bacterial lysates, extracellular polysaccharides, extracellular vesicles, surface proteins, metabolites, carbohydrates, enzymes, proteins, organic acids, lipids, vitamins, and complex molecules (Głowacka et al., 2024). Studies indicate that postbiotics contain chemically diverse molecules exhibiting various health-promoting properties and can intervene in neurodegenerative disease mechanisms (Aran et al., 2025; Kumar et al., 2025). Compared to live probiotics, postbiotics offer unique advantages, such as avoiding risks associated with live bacterial exposure, ensuring safety in individuals with compromised intestinal barriers, having extended shelf lives, and being easier to store and transport (Harat and Pourjafar, 2025; Martyniak et al., 2025). However, further research is required to address challenges such as identifying optimal postbiotic types, intervention timing, and duration for central nervous system disease prevention and treatment.

### 6.3 Fecal microbiota transplantation

Fecal microbiota transplantation (FMT) is a therapeutic approach that involves transplanting filtered fecal material from a healthy donor into the recipient’s intestinal tract (Yadegar et al., 2024). Studies have demonstrated that FMT holds significant potential for benefiting patients with Parkinson’s disease (PD) (Scheperjans et al., 2024). Preliminary research indicates that FMT can improve PD symptoms, particularly constipation, by modulating the composition of the intestinal microbiota, increasing beneficial bacteria and reducing harmful ones. Clinical trial results also show good tolerability and mild adverse reactions (Menozzi and Schapira, 2024). FMT has been shown to significantly alleviate intestinal microbiota metabolic disorders in PD mice, reduce intestinal inflammation and barrier damage, mitigate blood–brain barrier injury, lower the activation of microglia and astrocytes in the substantia nigra and striatum, inhibit neuroinflammation, and decrease components of the TLR4/TNF-α signaling pathway in both the gut and brain. These effects protect dopaminergic neurons and increase dopamine and serotonin levels in the striatum (Zhang X. et al., 2023). However, clinical trials of FMT for PD have not yet demonstrated consistent long-term efficacy. A research team in the Netherlands is further evaluating its long-term effects and safety (Vendrik et al., 2023). Additionally, studies have shown that FMT can reduce tau protein phosphorylation and Aβ levels in AD mice, enhance synaptic plasticity, and down-regulate the expression of inflammatory factors such as COX-2 and CD11b, thereby improving excessive neuroinflammation in AD mice (Sun et al., 2019). This suggests that FMT has potential application value in treating patients with central nervous system diseases.

### 6.4 Dietary interventions

Diet and nutritional supplements play a crucial role in shaping the diversity and function of the gut microbiota. They not only meet the host’s nutritional needs but also provide essential substrates for the gut microbiota. Studies have demonstrated that different components of the human diet can directly influence the composition and diversity of the gut microbiota (Kunnummal and Khan, 2024). Foods rich in phenols and antioxidants, such as fish, fresh fruits and vegetables, nuts, and berries, can mitigate the occurrence and progression of neurodegenerative diseases like Alzheimer’s by reducing oxidative stress and inflammatory responses in the brain (Chu et al., 2025). Conversely, a Western diet high in sugar and fat decreases the proportion of Bacteroidota (basonym Bacteroidetes), Bacillota (basonym Firmicutes), and Pseudomonadota (basonym Proteobacteria) in the gut microbiota, increasing intestinal and blood–brain barrier permeability, thereby affecting brain function (Jamar et al., 2021). The Mediterranean diet is recognized for its ability to modulate the composition and metabolism of the gut microbiota, acting as both an antioxidant and anti-inflammatory agent (Tan et al., 2022). Maraki et al. (2023) confirmed in a study of 1,047 participants that adherence to the Mediterranean diet can reduce the risk of pre-symptomatic PD. A systematic review of 52 studies also found that following the Mediterranean diet can decrease the incidence and clinical progression of PD. Certain dietary products, such as regular coffee consumption or foods rich in flavonoids, are associated with a reduced risk of developing PD in the future, while dairy products may increase this risk (Van Der Berg et al., 2024).

### 6.5 Nanotechnology

Due to their unique physicochemical properties, nanomaterials can mimic the functions of prebiotics and provide a suitable growth environment for beneficial gut bacteria (El-Dakroury et al., 2024). For instance, Shi Z. et al. (2024) developed a novel bionic oral drug, ZnPBA@YCW, by encapsulating zinc-doped Prussian blue analog (ZnPBA) nanozymes within a yeast cell wall (YCW) shell. After oral administration, this material targets the colon and specifically binds to *Escherichia coli* in the gut, releasing ZnPBA nanozymes to eliminate harmful bacteria while simultaneously scavenging reactive oxygen species (ROS) to suppress oxidative stress and inflammatory responses. Additionally, it regulates the intestinal microbiota of colitis mice by increasing the abundance of Bacillota (basonym Firmicutes) and *Ruminococcus* probiotics. Currently, most studies on the impact of nanomaterials on the intestinal microbiota are primarily limited to animal or *in vitro* experiments, with research on complex human systems remaining challenging. Moreover, the metabolism and elimination of nanomaterials pose significant safety concerns. Approximately 30–99% of nanoparticles in the bloodstream are taken up and accumulate in the liver, potentially causing chronic liver toxicity. Chen et al. (2019) found that oral administration of titanium dioxide nanoparticles increased the abundance of *Clostridium*, *Turicibacter*, and *Ruminococcus*, while significantly reducing the abundance of *Veillonella*, leading to intestinal microbiota dysbiosis. This dysbiosis further triggers oxidative stress and intestinal inflammation, resulting in significant changes in the levels of intestinal metabolites related to lipid metabolism and oxidative stress, such as N-acetylhistamine, glycerophosphocholine, and L-histidine. Therefore, the long-term safety of nanomaterials still requires further in-depth research and validation.

## 7 Conclusion

The concept of the “microbiota-gut-brain axis” has introduced a novel perspective to the study of CNS diseases. Through extensive research, we have gradually elucidated the intricate connections between the microbiota-gut-brain axis and CNS disorders. From a pathogenic standpoint, an imbalance in the gut microbiota can influence the CNS through multiple pathways. Firstly, when the intestinal barrier function is compromised, bacteria and their metabolites can enter the circulatory system, activating the immune system. The released inflammatory factors can cross the blood–brain barrier or travel via the vagus nerve, triggering inflammatory responses in the CNS. For instance, in Alzheimer’s disease and Parkinson’s disease, inflammatory responses are closely associated with neurodegeneration. Secondly, the gut microbiota can synthesize and modulate the metabolism of neurotransmitters such as serotonin and GABA. Alterations in these neurotransmitter levels are closely linked to the development of mental disorders like depression and anxiety. Additionally, the gut microbiota can interfere with the HPAA by influencing the neuroendocrine system, leading to dysregulation of the body’s stress response and exacerbating the progression of CNS diseases.

In terms of therapeutic approaches, several promising interventions have emerged based on the microbiota-gut-brain axis theory. The application of probiotics and prebiotics aims to restore the balance of the gut microbiota by supplementing beneficial bacteria and inhibiting the growth of harmful ones, thereby improving the intestinal microecology. Some clinical studies have shown that specific probiotic combinations can alleviate symptoms of anxiety and depression. FMT involves transferring the fecal microbiota from healthy individuals into the intestines of patients to restore normal gut microbiota. This approach has demonstrated positive therapeutic prospects in animal models of certain neurological diseases. Moreover, dietary interventions should not be overlooked. Foods rich in dietary fiber and polyphenols can promote the growth of beneficial microorganisms and positively impact the CNS.

However, numerous challenges remain in this field. On the one hand, while it is clear that the gut microbiota is related to CNS diseases, the specific microbial species and their metabolites that play key roles in disease development have not been fully identified, limiting the formulation of precise treatment strategies. On the other hand, most existing studies are primarily focused on animal experiments and small-scale clinical trials, lacking large-scale, long-term human studies to validate the safety and efficacy of intervention measures.

Looking to the future, in-depth exploration of the molecular mechanisms underlying the microbiota-gut-brain axis is crucial. The complex influence of the diversity of the intestinal microbiota (including archaea, fungi, and other microorganisms) on host physiology, particularly in the context of MGBA research, necessitates a multi-dimensional perspective. Archaea and fungi may indirectly modulate the MGBA via pathways such as methane production and short-chain fatty acid modification. Employing metagenomics, metabolomics, or cross-kingdom network analysis could enable a more comprehensive elucidation of microbial interaction mechanisms. Conducting large-scale, multi-center clinical studies and establishing standardized treatment protocols and evaluation systems will help determine the true efficacy and safety of microbiota-gut-brain axis-based treatments in CNS diseases. Additionally, interdisciplinary collaboration is vital. Joint efforts from experts in neuroscience, microbiology, immunology, and other relevant fields are expected to bring new breakthroughs in the prevention and treatment of CNS diseases, offering more hope to patients.
